# Mechanical traumatic injuries as a cause of death in free-ranging neotropical non-human primates living in anthropogenic matrices

**DOI:** 10.1007/s10329-026-01248-1

**Published:** 2026-03-09

**Authors:** Rafaela Magalhães Barros, Isabel Luana de Macêdo, Davi Emanuel Ribeiro de Sousa, Yasmin Nunes Godoy da Fonseca, Liz de Albuquerque Cerqueira, Gabriela Rodrigues de Toledo Costa, Liria Queiroz Luz Hirano, Márcio Botelho de Castro

**Affiliations:** 1 Veterinary Pathology and Forensic Laboratory, Faculty of Agronomy and Veterinary Medicine, University of Brasília, HVET - Via L4 Norte - Asa Norte, Brasília, Federal District 70910-900 Brazil; 2 Graduate Program in Animal Sciences, University of Brasília, Via L4 Norte - Asa Norte, Brasília, Federal District 70910-900 Brazil; 3 Environmental Health Surveillance Directorate of the Federal District, Brazilian Ministry of Health, AENW trecho 2 lote 4, Noroeste, Brasilia, Federal District 70684-831 Brazil; 4 Department of Wild Animals, Faculty of Agronomy and Veterinary Medicine, University of Brasília, HVET - Via L4 Norte - Asa Norte, Brasília, Federal District 70910-900 Brazil

**Keywords:** Marmoset, Callithrix penicillata, Trauma, Bone fracture, Predation, Urban environment

## Abstract

Traumatic injuries are a major cause of morbidity and mortality in free-ranging non-human primates (NHPs), often associated with anthropogenic pressures such as habitat loss, urbanization, and interactions with domestic animals. This study characterized some epidemiological, spatial, and pathological patterns of mechanical traumatic injuries in free-ranging non-human primates from anthropogenically altered areas of the Federal District. Of 696 necropsies performed, 215 cases (30.9%) involved mechanical trauma. Black-tufted marmosets (*Callithrix penicillata*) were the most affected species (90.2%), followed by capuchins (*Sapajus libidinosus*, 6.5%) and howler monkeys (*Alouatta caraya*, 3.3%). Adults were more frequently affected than juveniles, with no sex-related differences. Blunt trauma accounted for the majority of cases (82.3%), most commonly as polytrauma, with cranial involvement in 65.2% of animals. Carnivore predation was the leading cause of blunt-penetrating trauma (89.4%), while gunshot injuries were rare. Bone fractures were identified in 65.1% of cases, most often involving the skull and long bones, and internal injuries were frequent in the thoracic and abdominal cavities. These findings demonstrate that mechanical trauma represents a critical threat to NHPs in urban and peri-urban areas, where exposure to human infrastructure and domestic animals is heightened. The results underscore the need for mitigation strategies to reduce mortality and support the conservation of free-ranging primate populations in human-dominated landscapes.

## Introduction

In free-ranging non-human primates (NHPs), traumatic events are considered significant causes of injuries and death, often linked to anthropogenic factors (Ehlers et al. 2021). As human populations expand, NHPs increasingly face threats such as electrocution, vehicle collisions, intentional harm, and a number of threatening situations, particularly when foraging in urban and suburban environments (Pereira et al. 2020; La Chica et al. 2023; Barros et al. 2025).

Between 2001 and 2018, approximately 30% of global forest cover was lost due to human activities, with nearly half of this loss occurring in the Neotropics (i.e., Mexico, Central America, and South America), and Brazil being among the countries with the highest deforestation rates (Estrada et al. 2020). Road network expansion further exacerbates the risk of vehicle-related injuries to primates (Lavanya and Gokula 2023). Additionally, a Yellow Fever outbreak in NHPs and humans was likely related to an increase in trauma-related injuries in NHPs, attributed to human aggression driven by fear of disease transmission (Oliveira et al. 2022).

Proximity to residential areas contributes significantly to premature mortality in NHPs, largely through interactions with domestic predators, particularly dogs and cats (Gordo et al. 2013), and has been identified as a significant cause of trauma-related deaths in NHPs (Ehlers et al. 2021; Bicca-Marques et al. 2020). In howler monkeys, intraspecific aggression has been associated with immigration events, often triggered by habitat loss and fragmentation (Cristóbal-Azkarate et al. 2004). Among trauma-related causes, electrocution is particularly notable, especially in free-ranging NHPs inhabiting anthropized regions of Central Brazil (Pereira et al. 2020; Barros et al. 2025), indicating that some primate species may be unable to fully adapt to the hazards of rapidly urbanizing and fragmented landscapes. Maladaptive behaviors resulting from living in varying degrees of human-altered environments have been reported in free-ranging macaques in India and Thailand (Dhawale et al. 2020; Luncz et al. 2017).

The diagnosis of traumatic injuries in wildlife can serve as a valuable indicator of environmental disturbance and habitat alteration, providing essential insights into the ecological consequences of human activities (Barros et al. 2025). The present study aims to characterize some epidemiological, spatial, and pathological patterns of mechanical traumatic injuries in free-ranging NHPs inhabiting mainly anthropogenically altered landscapes of the Federal District.

## Materials and methods

This study comprised a retrospective analysis of necropsy records of NHPs from the Laboratory of Veterinary Pathology and Forensics at the University of Brasília (LVPF-UnB), covering the period from January 2018 to December 2023. Only cases involving mechanical trauma were included. Data evaluated included species, sex, age group (juvenile or adult) (Decanini and Macedo 2008), date of necropsy, and gross findings. The georeferenced locations of deaths or collection sites of injured animals, when available, were used to generate a distribution map of traumatic injury cases, using QGIS software (version 3.40.7 – Bratislava).

Dates of NHP deaths were organized by year, month, season (spring, summer, autumn, or winter), and precipitation period (rainy season: October to April; dry season: May to September). As a standard protocol, all necropsies included complete removal of the skin to expose the subcutaneous tissues and lesions, along with photographic and written documentation of the procedure. Each case was carefully re-evaluated based on photographic and necropsy records. Cases lacking photographic documentation were excluded.

The LVPF is an Official Regional Reference Laboratory under the Brazilian Ministry of Health, supporting the diagnosis and surveillance of epizootics in NHPs within the framework of the National Control Program for Yellow Fever and other zoonotic diseases. In the Federal District, NHPs are most often reported dead or injured by residents, who then notify the Environmental Military Police or the Local Health Surveillance Service. These agencies are responsible for retrieving animals from urban and peri-urban areas and forwarding them for medical care or necropsy (Pereira et al. 2020; Barros et al. 2025).

Mechanical traumatic injuries were classified as blunt and blunt-penetrating. Blunt trauma was further subdivided into localized (a single injury site) and polytrauma (two or more injury sites). Blunt-penetrating injuries were classified as either carnivore predation or ballistic projectile wounds. Cranioencephalic trauma was categorized as localized (head only) or associated with polytrauma (multiple injury sites including the head).

Traumatic lesion distribution was assessed by anatomical region, including the head, neck, trunk, limbs, and tail. Injuries were further mapped to the thoracic limbs (shoulder, arm, forearm, and hand), pelvic limbs (pelvis, thigh, leg, and foot), and the trunk (thorax, abdomen, and pelvis). The number of fractures per animal, affected bones, and fracture types (complete or incomplete; simple or comminuted; open or closed) were recorded. Injuries to body cavities and internal organs were classified into central nervous system, thoracic, and abdominal/pelvic lesions.

Frequencies (%) were calculated for all variables, including affected NHP species and genera, sex, age categories, and temporal factors associated with trauma (month, year, season, and rainfall period). A descriptive analysis was conducted to determine the frequency distribution of gross findings, trauma classifications, and anatomical distribution of injuries. When applicable, frequency comparisons were performed using Fisher’s Exact Test or Chi-Square Test in GraphPad Prism 8.0.

## Results

**Fig. 1:**
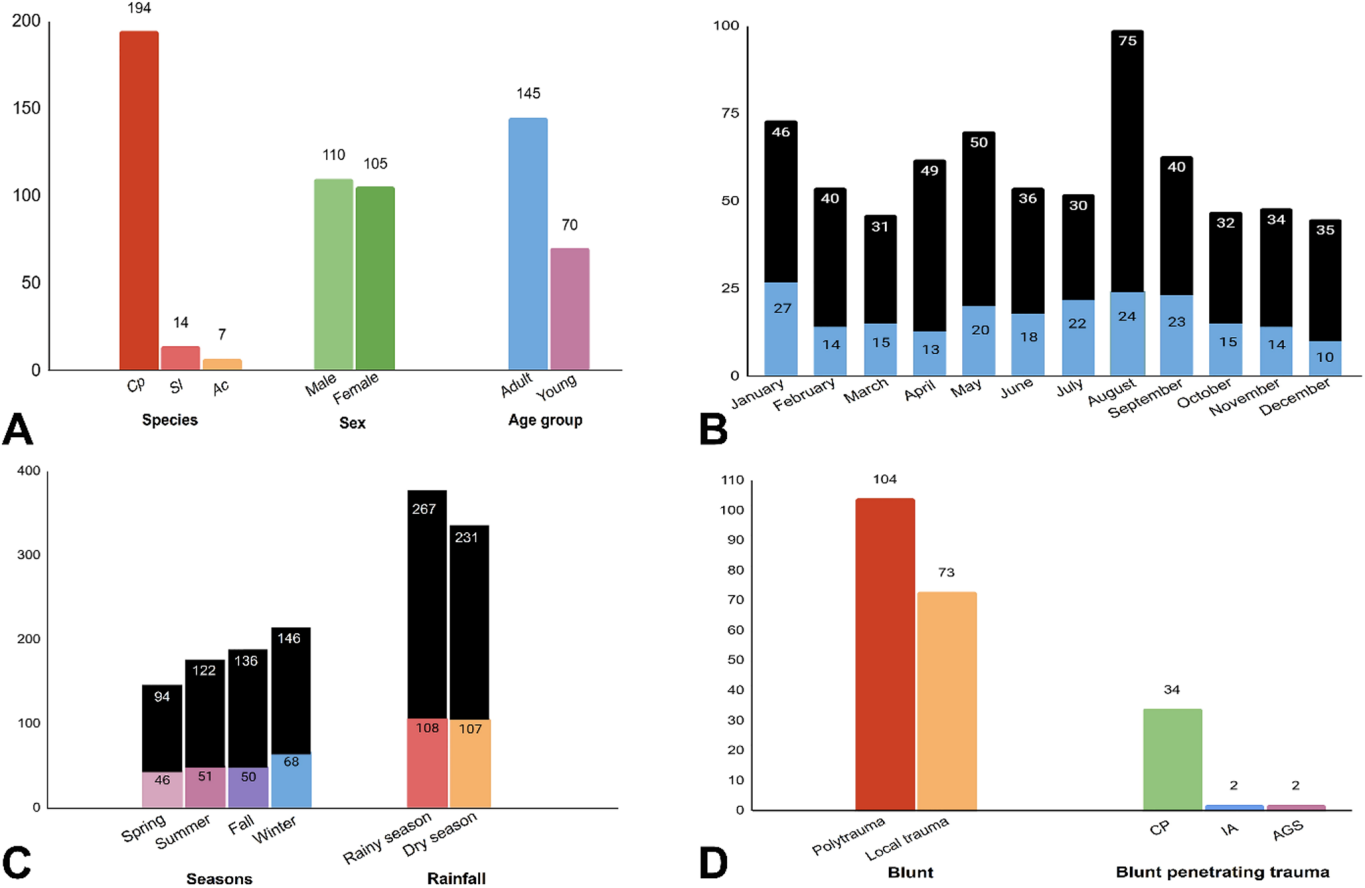
General and pathological data on non-human primates (NHPs) victims of mechanical trauma injuries. (**A**) Species, sex, and age group of affected animals (*n* = 215). CP *Callithrix penicillata.* SL *Sapajus libidinosus.* AC *Alouatta caraya*. (**B**) Total number of NHPs affected by mechanical trauma (blue, *n* = 215) and other causes of death (black, *n* = 481) aggregated monthly from 2019 to 2022. (**C**) The monthly distribution of traumatic injuries and their frequencies across annual rainy and dry periods, and climate seasons (p˃0,05). (**D**) Frequencies of polytrauma (*n* = 104) and localized trauma for 41.2% (*n* = 73/177) of blunt injuries; and blunt-penetrating injuries including carnivore predation (CP, *n* = 34), intra-species aggression (IA, *n* = 2) and air gun shooting injury (AGS, *n* = 2)

Between 2018 and 2023, a total of 713 necropsies were performed on NHPs at the LPPV-UnB, of which 232 exhibited mechanical traumatic injuries. Seventeen cases of trauma were excluded due to incomplete data, resulting in 696 necropsies eligible for evaluation. Ultimately, a total of 215 animals (30.9%) were included in the study. The most affected NHP species was *Callithrix penicillata* (90.2%, *n* = 194/215), followed by *Sapajus libidinosus* (6.5%, *n* = 14/215) and *Alouatta caraya* (3.3%, *n* = 7/215) (*p* < 0,05) (Fig. 1A).

No sex-related difference was observed (53.6%, *n* = 110/205 males vs. 46.4%, *n* = 105/215 females, p˃0,05), while adults were more affected (67.4%, *n* = 145/215, *p* < 0,05) (Fig. 1A). The monthly distribution of traumatic injuries (Fig. 1B) as well as their frequencies across annual rainy and dry periods, and climate seasons (Fig. 1C) showed no significant differences (p˃0,05).

Blunt trauma was the most frequent (82.3%, *n* = 177/215) cause of injuries, followed by blunt-penetrating trauma (17.7%, *n* = 38/215) (Fig. 1D). Polytrauma accounted for 58.8% (*n* = 104/177), and localized trauma for 41.2% (*n* = 73/177) of blunt injuries (Fig. 1D). Of the animals with polytrauma, 62.5% (65/104) had concurrent head trauma. Among localized traumas, 69.8% (*n* = 51/73) affected the head only, followed by the abdomen (11.0%, *n* = 8/73), legs (5.5%, *n* = 4/73), thorax (4.1%, *n* = 3/73), lumbosacral region (2.7%, *n* = 2/73), thighs (2.7%, *n* = 2/73), pelvis (1.4%, *n* = 1/73), arm (1.4%, *n* = 1/73), and forearm (1.4%, *n* = 1/73).

**Fig. 2:**
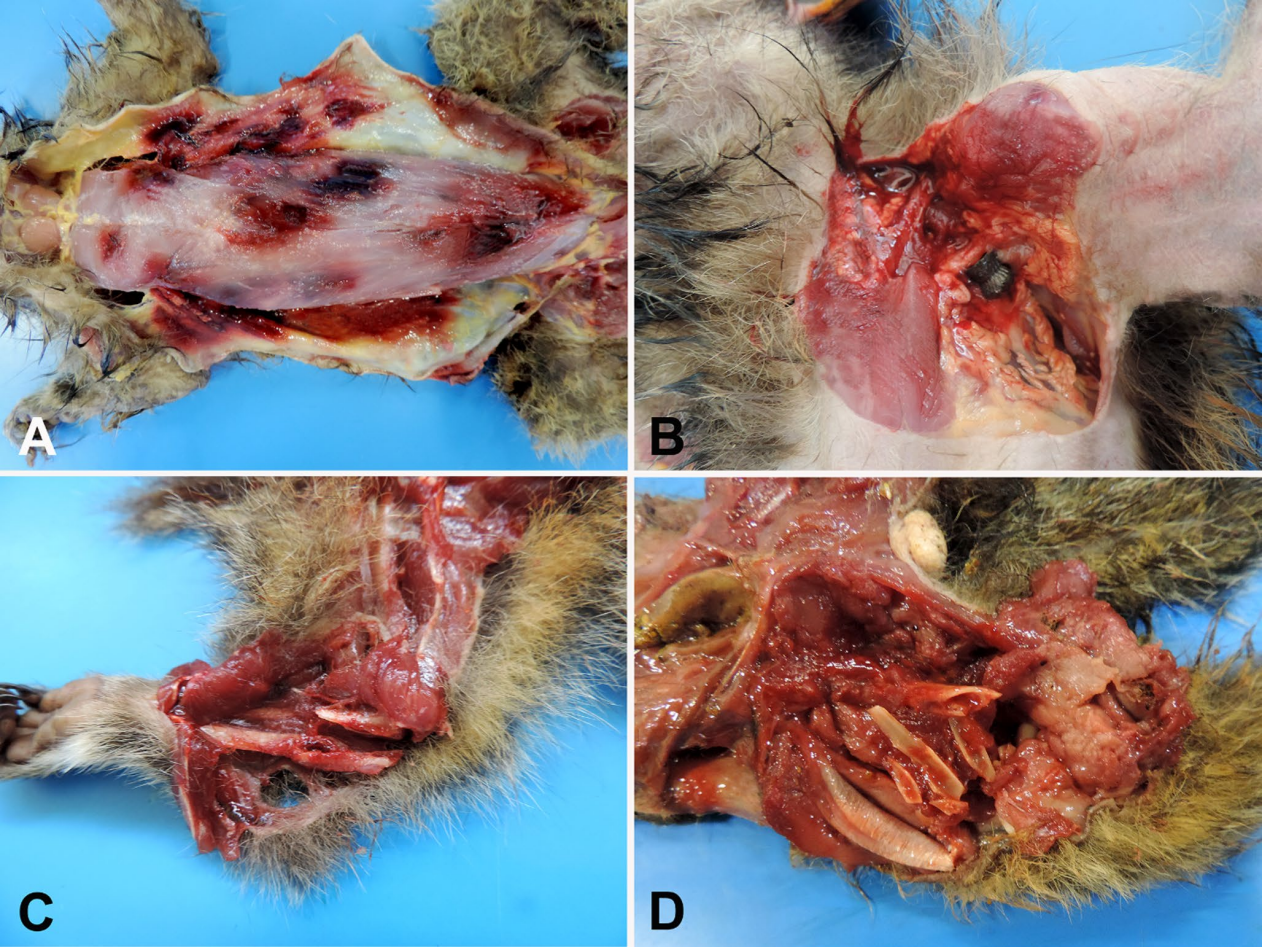
Gross lesions in NHPs victims of mechanical trauma injuries. (**A**) Blunt-penetrating injuries due to carnivore predation in a marmoset. (**B**) Air gun shooting injury in a *C. penicillata*. (**C**) A marmoset with complete bone fractures of the radius and ulna. (**D**) Complete comminuted fracture involving the femur in a *C. penicillata*

**Fig. 3:**
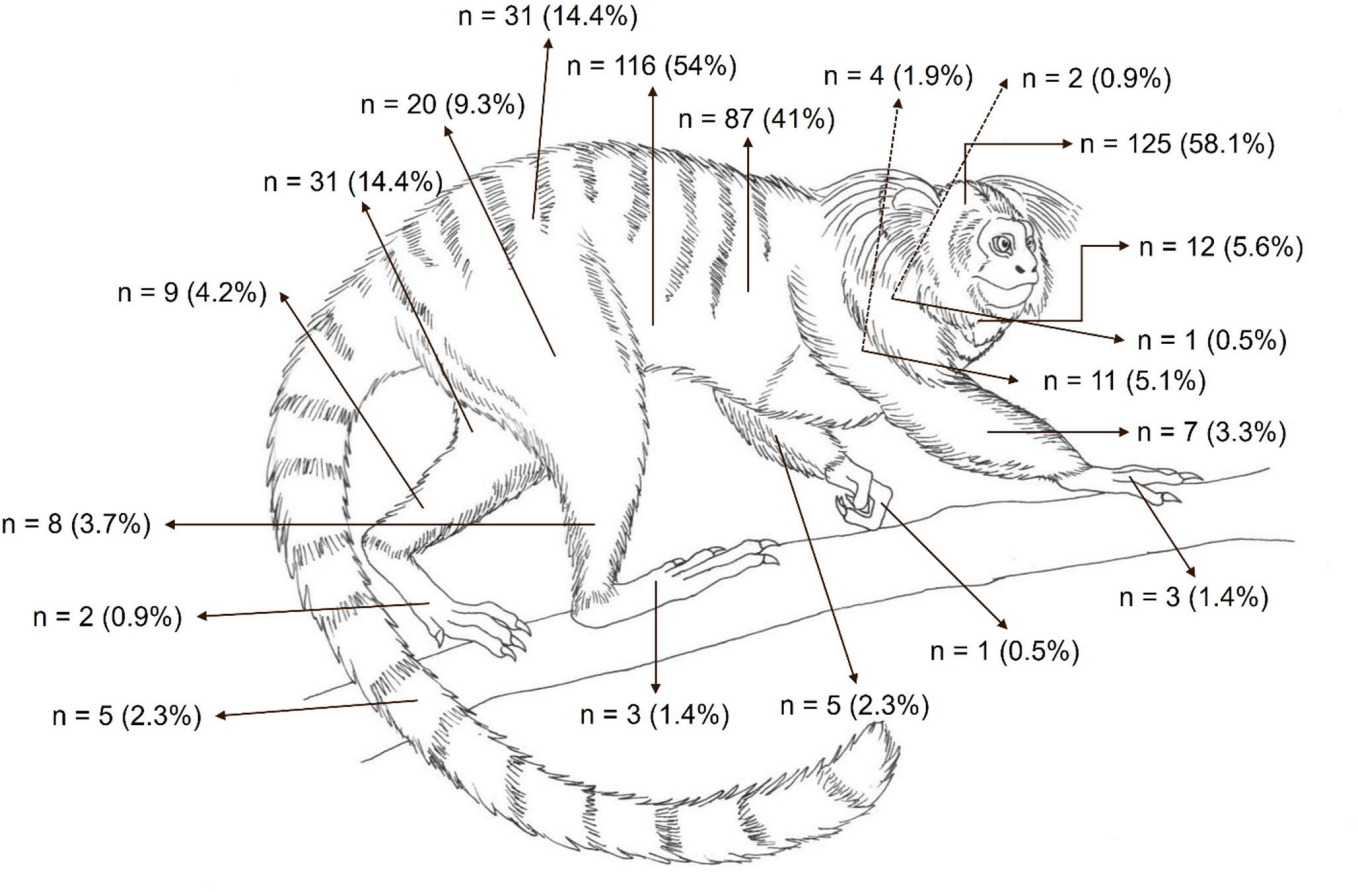
Diagram illustrating the anatomical distribution and frequencies (%) of mechanical traumatic injuries in NHPs

**Fig. 4:**
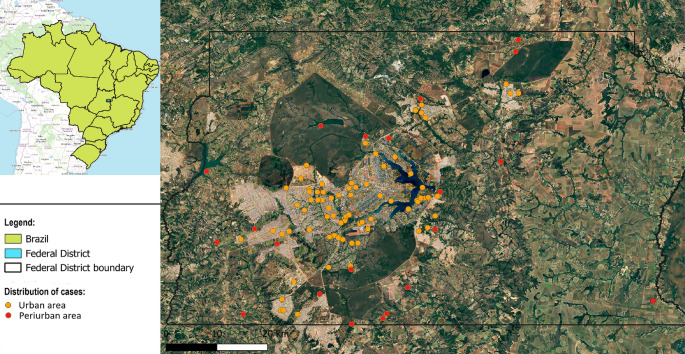
Spatial distribution of traumatic injuries in NHPs (*n* = 122) in the Federal District, Brazil

**Fig. 5:**
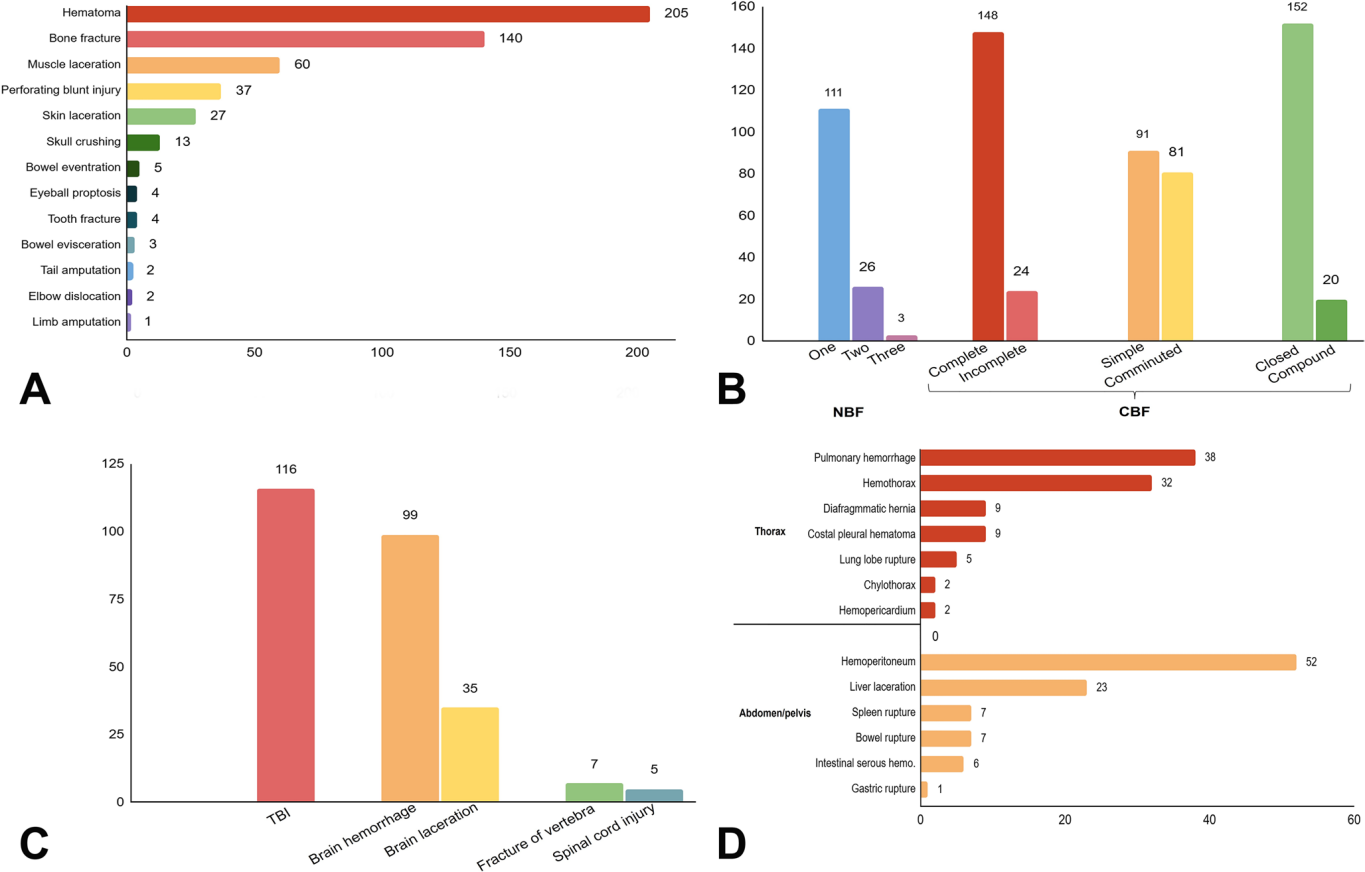
Mechanical trauma injuries in NHPs (number of animals). (**A**) Trauma-associated lesions in NHPs. (**B**) Number of bone fractures (NBF) by animal and classification of bone fractures (CBF) by type. (**C**) Injuries affecting the nervous system and related tissues (TBI: traumatic brain injury). (**D**) Injuries affecting thoracic, abdominal and pelvic organs and tissues

Carnivore predation was the most common cause (89.4%, *n* = 34/38) of blunt-penetrating injuries (Fig. 2A), followed by witnessed intra-species aggression in two howler monkeys (5.3%, *n* = 2/38) and air gun shooting injury (5.3%, *n* = 2/38) (Figs. 1D and 2B). No penetrating injuries were observed in this study. The most frequently affected body regions by mechanical trauma were the head (58.1%, *n* = 125/215) and abdomen (53.9%, *n* = 116/215), followed by the thorax (40.4%, *n* = 87/215), pelvic limbs (33.9%, *n* = 73/215), thoracic limbs (15.8%, *n* = 34/215), pelvis (14.4%, *n* = 31/215), neck (5.6%, *n* = 12/215), and tail (2.3%, *n* = 5/215) (Fig. 3). The spatial distribution of traumatic injuries among georeferenced NHP cases (*n* = 122) was concentrated primarily in urban and peri-urban areas, as illustrated in Fig. 4. Figure 5A presents the major mechanical trauma-related tissue lesions identified in the NHPs.

**Fig. 6:**
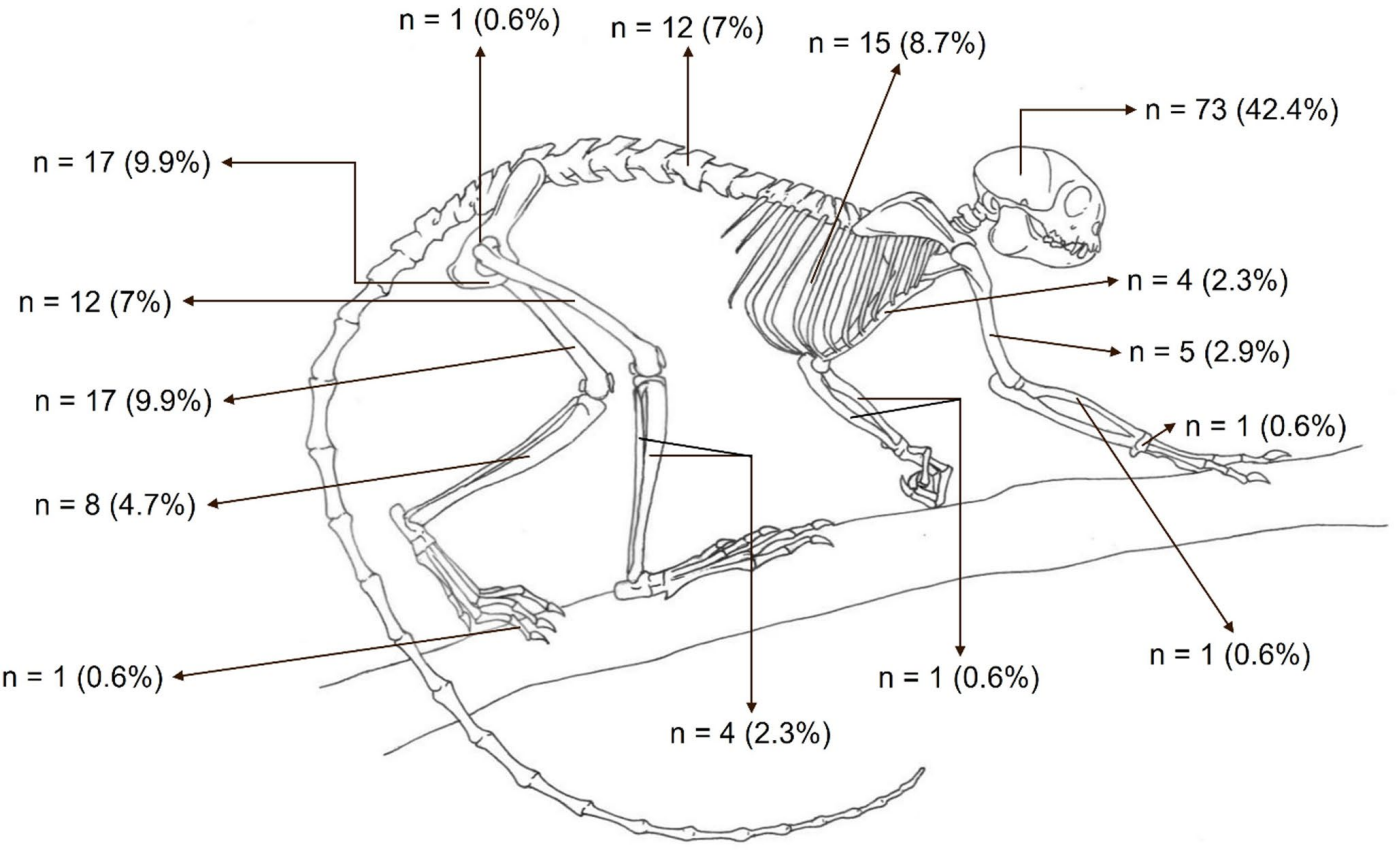
Diagram illustrating the anatomical distribution and frequencies (%) of bone fractures in NHPs (*n* = 172)

**Fig. 7:**
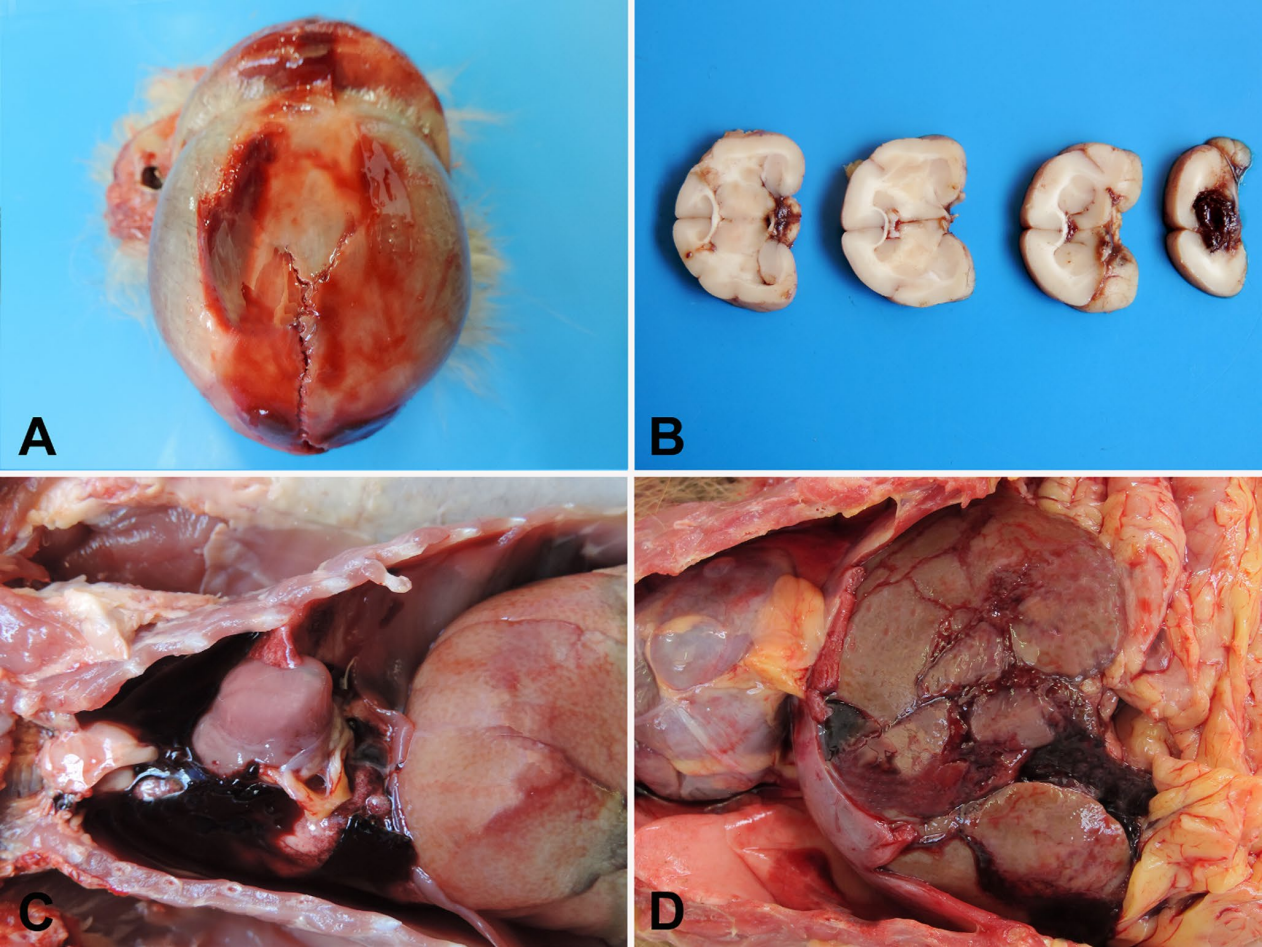
A. Gross lesions in NHPs victims of mechanical trauma injuries. (**A**) A *C. penicillata* with cranial fracture with surrounding hematoma. (**B**) Transverse sections of the brain showing hemorrhage in a marmoset with craniocerebral trauma. (**C**) A marmoset with hemothorax. (**D**) Hepatic laceration in a *C. penicillata*

Bone fractures were documented in 65.1% (140/215) of the NHPs included in this study. Of these, 79.3% (111/140) exhibited a single fracture, 18.6% (26/140) had fractures in two bones, and 2.1% (3/140) exhibited fractures in three distinct bones (Fig. 5B). The types of bone fractures are detailed in Fig. 5B; examples are shown in Figs. 2C, D, and their anatomical distribution is illustrated in Fig. 6. Of the 177 animals that sustained blunt force trauma, 65.2% (*n* = 116/177) exhibited cranial involvement (Fig. 7A), frequently with brain injury (Fig. 7B), and 3.9% (*n* = 7/177) had vertebral fractures (Fig. 5C).

Thoracic trauma was observed in 39.5% (*n* = 85/215) of cases, followed by abdominal injuries in 54.0% (*n* = 116/215), and pelvic injuries in 14.4% (*n* = 31/215). The most frequent thoracic lesions were pulmonary hemorrhage (17.7%, *n* = 38/215) and hemothorax (14.9%, *n* = 32/215) (Fig. 7C). Within the abdominal and pelvic cavities, hemoperitoneum was noted in 14.9% (*n* = 32/215), and hepatic lacerations in 10.7% (*n* = 23/215) (Fig. 7D). Additional internal injuries are presented in Fig. 5D.

## Discussion

Natural environments can undergo profound anthropogenic transformations, posing significant impacts and threats to local wildlife. In this context, the present study demonstrated that free-ranging NHPs, particularly those inhabiting urban and peri-urban areas, were commonly affected by mechanical trauma as a cause of death, accounting for 30.2% of cases. In small-scale studies in southern and northeastern Brazil, traumatic injuries have been similarly described as the leading cause of death among free-ranging NHPs, and strongly associated with environmental anthropization (Ehlers et al. 2021; Dias et al. 2022). In addition to traumatic mechanical injuries, recent studies reported that electrocution accounted for 11.0 to 16.5% of fatalities among similar NHP populations living in the same region of this study and under analogous environmental conditions (Pereira et al. 2020; Barros et al. 2025). Therefore, nearly half of the NHPs living in the Federal District and surrounding regions likely have been dying due to mechanical or physical traumatic injuries.

Adult black-tufted marmosets (*C. penicillata*) were the most frequently affected species, which may be explained by their higher abundance in the study area compared to capuchim (*S. libidinosus*) and howler monkeys (*A. caraya*), as previously observed (Pereira et al. 2020; Barros et al. 2025). Additionally, howler monkeys tend to avoid human presence and prefer more preserved natural habitats (Oliveira et al. 2022).

Traumatic injuries occurred throughout the year, with no evidence of seasonality, underscoring the constant hazards faced by these animals in urbanized environments. Blunt force trauma, particularly polytrauma (58.8%), was the most frequently observed injury type. In many cases, the etiology of these mechanical injuries could not be determined, primarily because of the non-specific nature of the morphological alterations and the absence of clinical histories for free-ranging animals. The severity of such injuries is influenced by the magnitude of the mechanical energy involved, the anatomical site and nature of the trauma, and the intrinsic resistance of the affected tissues (Merk et al. 2013). While arboreal Neotropical NHPs are evolutionary adapted to natural forested habitats (Rangel et al. 2013), and urban landscapes and man-made structures expose them to a variety of threats, including vehicular collisions, accidental falls, predation by domestic animals, and intentional human-inflicted harm (Ehlers et al. 2021; Dias et al. 2022), resembling the distribution and severity of lesions documented in this study.

Subcutaneous and muscular hematomas were detected in virtually all NHPs victims of trauma (98.4%), and they were a result of the tissue damage caused by the direct mechanical force hitting living tissues (Munro and Munro 2008). As expected, blunt trauma also promoted bone fractures in most animals (79.3%) distributed across the body, although some anatomical sites, such as the skull and long bones, were more commonly affected. These findings are consistent with reports in catarrhine primates, particularly great apes (Lovell 1991), but similar detailed records are still lacking in neotropical NHPs (Ehlers et al. 2021; Dias et al. 2022).

In this study, pelvic limbs (33.9%) were more frequently affected than thoracic limbs (15.8%), a pattern also reported in a colony of free-ranging rhesus monkeys in Puerto Rico (Buikstra 1975), but contrasting with the higher incidence of forelimb fractures in East African chimpanzees, which has been linked to interspecific violence (Jurmain 1989). Complete, closed, simple, or comminuted fractures were the most common bone injuries observed in NHPs from the Federal District and surrounding areas, typically associated with diaphyseal lesions in long bones (Decamp et al. 2016). Conversely, high-energy and high-velocity trauma, such as vehicular collisions, more often produce severe soft tissue damage and extensively fragmented fractures (Millard and Weng 2014), particularly involving the pubic bones (Merk et al. 2013).

Eventrations and eviscerations were observed in a few cases evaluated and are considered uncommon outcomes of blunt abdominal trauma (Peterson et al. 2015; Ibrahim et al. 2020). In NHPs, these injuries may result from falls from height, such as during leaps between trees (Kumar and Raj 2012). Similar to other trauma patterns, amputations were rare in evaluated animals, and typically are related to tangential or oblique blunt force impacts, which can shear the skin and/or soft tissues from the underlying fascia or bone (Merk et al. 2013).

A key finding of this study, with direct clinical relevance for the management of NHPs in wildlife rescue services, was the high frequency of head trauma (65.2%), which represented one of the leading causes of death, consistent with reports in human patients (Capizzi and Gutierrez 2020; Schmidt 2020). Elevated rates of head injuries have also been documented in free-ranging marmosets (Oliveira et al. 2022), although in other NHP species, trauma more frequently affects the hands, feet, or tail (Lovell 1991). Among nervous system injuries, spinal cord lacerations were identified in a few cases, typically in association with vertebral fractures and polytrauma, suggesting a likely origin from falls from considerable heights.

Blunt trauma of undetermined origin was the predominant cause of thoracic injury, most frequently resulting in pulmonary hemorrhage and hemothorax, and less commonly in diaphragmatic hernia, lung lobe rupture, chylothorax, and hemopericardium. Pulmonary contusions, typically arising from high-energy impacts, cause parenchymal destruction and alveolar hemorrhage (Merk et al. 2013; Požgain et al. 2018). Likewise, hemothorax may develop secondary to injury of the intercostal vessels, pulmonary lacerations, or rupture of major mediastinal vessels (Tsai et al. 1999; Yu et al. 2017), leading to hypovolemia, pulmonary atelectasis, and respiratory distress (Dogrul et al. 2020). The occurrence of diaphragmatic rupture, pulmonary lacerations, hemopericardium, and chylothorax, an uncommon finding in NHPs (Barros et al. 2024), underscores the diverse range of thoracic lesions that can significantly contribute to mortality in trauma-affected individuals.

Within the abdominal cavity, hemoperitoneum was identified in approximately 15% of animals, most often associated with hepatic laceration and/or splenic rupture, lesions that can precipitate hypovolemic shock and death (Merk et al. 2013). Although intestinal and gastric ruptures were recorded in only a few cases, such injuries markedly increase mortality risk owing to the high likelihood of peritonitis and sepsis (Faria et al. 2012; Özpek et al. 2024).

Blunt penetrating trauma was documented in 17.8% of cases, most commonly resulting from carnivore predation, as previously reported (Ehlers et al. 2021), and less frequently from intraspecific aggression, both resulting in severe hemorrhage and extensive tissue damage. Intraspecific aggression has been recorded in different NHP species in Southeastern and Southern regions (Talebi et al. 2009; Ehlers et al. 2021), and was documented in this study as agonistic encounters between howler monkeys resulting in polytrauma. Air gun injuries, although rare in this series, produced distinctive blunt penetrating fatal trauma characterized by extensive soft tissue damage and marked hemorrhage (Prat et al. 2017).

The set of our findings highlights the dangers posed by anthropogenic environments to free-ranging NHPs. Mechanical trauma has emerged as a major cause of death, particularly among populations inhabiting urban and peri-urban areas, where contact with human infrastructure and domestic animals is frequent. From a conservation perspective, the expansion of anthropogenic environments and their associated hazards may disrupt the natural locomotor behaviors, foraging strategies, feeding patterns, territorial interactions, and dispersal routes of NHP species, potentially allowing maladaptive behavioral choices that increase their vulnerability to fatal injury. These results underscore the pressing need for targeted mitigation strategies, such as wildlife-friendly infrastructure design, habitat restoration, and public education, to reduce mortality, promote coexistence, and ensure the long-term viability of NHP populations in increasingly human-dominated environments.

## References

[CR1] Barros RM, Macêdo IL, Sousa DER, Hirano LQL, Paludo GR, Castro MB (2024) Traumatic chylothorax in urbanized free-ranging black-tufted marmosets (*Callithrix penicillata*). J Med Primatol 53(1):e12660 37394724 10.1111/jmp.12660

[CR2] Barros RM, Macêdo IL, Sousa DER, Cerqueira LA, Fonseca YNG, Sousa ALV, Santos AD, de Melo CB, Castro MB (2025) Electrocutions in free-ranging platyrrhine non-human primates: diagnostic features for a threatening condition. Am J Primatol 87(4):e70039. 10.1002/ajp.70039 40253702 10.1002/ajp.70039 PMC12009613

[CR3] Bicca-Marques JC, Chaves ÓM, Hass GP (2020) Howler monkey tolerance to habitat shrinking: lifetime warranty or death sentence? Am J Primatol 82(4):e23089. 10.1002/ajp.23089 31912561 10.1002/ajp.23089

[CR4] Buikstra JE (1975) Healed fractures in *Macaca mulatta*: age, sex, and symmetry. Fol Primatol 23(1–2):140–148. 10.1159/000155667 10.1159/000155667 1140749

[CR5] Capizzi A, Woo J, Verduzco-Gutierrez M (2020) Traumatic brain injury: an overview of epidemiology, pathophysiology, and medical management. Med Clin North Am 104(2):213–238. 10.1016/j.mcna.2019.11.001 32035565 10.1016/j.mcna.2019.11.001

[CR6] Cristóbal-Azkarate J, Dias PAD, Veà JJ (2004) Causes of intraspecific aggression in *Alouatta palliata mexicana*: evidence from injuries, demography, and habitat. Int J Primatol 25(4):939–953. 10.1023/B:IJOP.0000029130.10312.63

[CR7] Decamp CE, Johnston AS, Déjardin LM, Schaefer SL (2016) Fractures of the tibia and fibula. Brinker, Piermattei and Flo’s Handbook of Small Animal Orthopedics and Fracture Repair, 5ª ed. Saunders Elsevier, Philadelphia, pp 670–706. 10.1016/C2009-0-64185-4

[CR8] Decanini DP, Macedo RH (2008) Sociality in *Callithrix penicillata*: II. Individual strategies during intergroup encounters. Int J Primatol 29:627–639. 10.1007/s10764-008-9265-6

[CR9] Dhawale AK, Kumar MA, Sinha A (2020) Changing ecologies, shifting behaviours: behavioural responses of a rainforest primate, the lion-tailed macaque *Macaca silenus*, to a matrix of anthropogenic habitats in southern India. PLoS One 15(9):e0238695. 10.1371/journal.pone.0238695 32966281 10.1371/journal.pone.0238695 PMC7511024

[CR10] Dias RFF, Gonçalves SRF, Barretto MLM, Albuquerque PPF, Silva Junior VA, Barros MR, Oliveira JB, Leal SG, Oliveira AAF (2022) Postmortem findings in non-human primates from Pernambuco, Brazil. Res Soc Dev 11(4):e12511427276. 10.33448/rsd-v11i4.27276

[CR11] Dogrul BN, Kiliccalan I, Asci ES, Peker SC (2020) Blunt trauma related chest wall and pulmonary injuries: an overview. Chin J Traumatol 23(3):125–138. 10.1016/j.cjtee.2020.04.003 32417043 10.1016/j.cjtee.2020.04.003 PMC7296362

[CR12] Ehlers LP, Slaviero M, Bianchi MV, De Mello LS, De Lorenzo C, Surita LE, Alievi MM, Driemeier D, Pavarini SP, Sonne L (2021) Causes of death in neotropical primates in Rio Grande do Sul State, Southern Brazil. J Med Primatol 51(2):85–92. 10.1111/jmp.12557 34862608 10.1111/jmp.12557

[CR13] Estrada A, Garber PA, Chaudhary A (2020) Current and future trends in socio-economic, demographic and governance factors affecting global primate conservation. PeerJ 21(8):e9816. 10.7717/peerj.9816 10.7717/peerj.9816 PMC7444509 32884865

[CR14] Faria GR, Almeida AB, Moreira H, Barbosa E, Correia-da-Silva P, Costa-Maia J (2012) Prognostic factors for traumatic bowel injuries: killing time. World J Surg 36(4):807–812. 10.1007/s00268-012-1458-7 22350477 10.1007/s00268-012-1458-7

[CR15] Gordo M, Calleia FO, Vasconcelos SA, Leite JJF, Ferrari SF (2013) The challenges of survival in a concrete jungle: Conservation of the pied tamarin (Saguinus bicolor) in the urban landscape of Manaus, Brazil. In: Chapman CA, Marsh LK (eds) Primates in fragments: Complexity and resilience. Springer, New York, pp 357–370. 10.1007/978-1-4614-8839-2

[CR16] 10.1111/jmp.12660

[CR17] Ibrahim AH, Osman AJ, Alarfaj MA, Alzamil AM, Abahussain MA, Alghamdi H (2020) Case report: evisceration of abdomen after blunt trauma. Int J Surg Case Rep 72:207–211. 10.1016/j.ijscr.2020.05.037 32544830 10.1016/j.ijscr.2020.05.037 PMC7298532

[CR18] Jurmain R (1989) Trauma, degenerative disease, and other pathologies among the Gombe chimpanzees. Am J Phys Anthropol 80(2):229–237. 10.1002/ajpa.1330800211 2801915 10.1002/ajpa.1330800211

[CR19] Kumar V, Raj A (2012) Surgical management of unilateral inguinoscrotal hernia in a male rhesus macaque. J Vet Sci Technol 1(1):1–4

[CR20] La Chica AG, Oklander LI, Kowalewski MM, Fernandez-Duque E (2023) Human and non-human primate coexistence in Argentina: conflicts and solutions. Animals (Basel) 13(21):3331. 10.3390/ani13213331 37958086 10.3390/ani13213331 PMC10648367

[CR21] Lavanya R, Gokula V (2023) Impact of road traffic on Mysore slender loris *Loris lydekkerianus lydekkerianus* in Keelaveliyur, Tamil Nadu, India. Uttar Pradesh J Zool 44(5):25–34

[CR22] Lovell NC (1991) An evolutionary framework for assessing illness and injury in non-human primates. Am J Phys Anthropol 34(S13):117–155. 10.1002/ajpa.1330340608

[CR23] Luncz LV, Svensson MS, Haslam M, Malaivijitnond S, Proffitt T, Gumert M (2017) Technological response of wild macaques (*Macaca fascicularis*) to anthropogenic change. Int J Primatol 38(5):872–880. 10.1007/s10764-017-9985-6 29056799 10.1007/s10764-017-9985-6 PMC5629225

[CR24] Merck MD, Miller DM, Reisman RW, Maiorka PC (2013) Blunt Force Trauma. In: Merck MD (ed) Veterinary Forensics: Animal Cruelty Investigation, 2nd ed. Wiley-Blackwell, Ames, pp 111–121

[CR25] Millard RP, Weng HY (2014) Proportion of and risk factors for open fractures of the appendicular skeleton in dogs and cats. J Am Vet Med Assoc 245(6):663–668. 10.2460/javma.245.6.663 25181270 10.2460/javma.245.6.663

[CR26] Munro HM, Munro R (2008) Wounds and Injuries. Animal abuse and unlawful killing: forensic veterinary pathology. Elsevier, Philadelphia, pp 30–47

[CR27] Oliveira AR, Santos DO, Lucena FP, Mattos SA, Carvalho TP, Costa FB, Moreira LGA, Vasconcelos IMA, Paixão TA, Santos RL (2022) Non-thrombotic pulmonary embolism of brain, liver, or bone marrow tissues associated with traumatic injuries in free-ranging neotropical primates. Vet Pathol 59(3):482–488. 10.1177/03009858221075595 35130802 10.1177/03009858221075595

[CR28] Özpek A, Yıldırak MK, Ezberci F (2024) Hollow viscus injury due to blunt abdominal trauma: a tertiary trauma center experience. Turk J Trauma Emerg Surg 30(2):123–128. 10.14744/tjtes.2024.67249 10.14744/tjtes.2024.67249 PMC10977501 38305660

[CR29] Pereira AABG, Dias B, Castro SI, Landi MFA, Melo CB, Wilson TM, Costa GRT, Passos PHO, Romano AP, Szabó MPJ, Castro MB (2020) Electrocutions in free-living black-tufted marmosets (*Callithrix penicillata*) in anthropogenic environments in the Federal District and surrounding areas, Brazil. Primates 61(2):321–329. 10.1007/s10329-019-00760-x 31564005 10.1007/s10329-019-00760-x

[CR30] Peterson NW, Buote NJ, Barr JW (2015) The impact of surgical timing and intervention on outcome in traumatized dogs and cats. J Vet Emerg Crit Care 25(1):63–75. 10.1111/vec.12279 10.1111/vec.12279 25605629

[CR31] Požgain Z, Kristek D, Lovrić I, Kondža G, Jelavić M, Kocur J, Danilović M (2018) Pulmonary contusions after blunt chest trauma: clinical significance and evaluation of patient management. Eur J Trauma Emerg Surg 44(5):773–777. 10.1007/s00068-017-0876-5 29167928 10.1007/s00068-017-0876-5

[CR32] Prat NJ, Daban JL, Voiglio EJ, Rongieras F (2017) Wound ballistics and blast injuries. J Visc Surg 154(Suppl 1):S9–S12. 10.1016/j.jviscsurg.2017.07.005 28941569 10.1016/j.jviscsurg.2017.07.005

[CR33] Rangel CH, Adler JGV, Heliodor GC, Santos JA, Verona CE (2013) Relato de caso de morte por agressão entre macacos prego *Sapajus nigritus* (*Primates: Cebidae*) no Jardim Botânico do Rio de Janeiro. Neotrop Primates 20(1):48–52. 10.1896/044.020.0108

[CR34] Schmidt U, Oramary D, Kamin K, Buschmann CT, Kleber C (2020) Synergistic effects of forensic medicine and traumatology: comparison of clinical diagnosis autopsy findings in trauma-related deaths. World J Surg 44(4):1137–1148. 10.1007/s00268-019-05347-7 31933040 10.1007/s00268-019-05347-7

[CR35] Talebi MG, Beltrão-Mendes R, Lee PC (2009) Intra-community coalitionary lethal attack of an adult male southern muriqui (*Brachyteles arachnoides*). Am J Primatol 71(10):860–867. 10.1002/ajp.20713 19489067 10.1002/ajp.20713

[CR36] Tsai FC, Chang YS, Lin PJ, Chang CH (1999) Blunt trauma with flail chest and penetrating aortic injury. Eur J Cardio-Thorac Surg 16(3):374–377. 10.1016/s1010-7940(99)00230-4 10.1016/s1010-7940(99)00230-4 10554864

[CR37] Yu H, Isaacson AJ, Burke CT (2017) Management of traumatic hemothorax, retained hemothorax, and other thoracic collections. Curr Trauma Rep 3:181–189. 10.1007/s40719-017-0101-3

